# *Escherichia coli* O-Antigen Gene Clusters of Serogroups O62, O68, O131, O140, O142, and O163: DNA Sequences and Similarity between O62 and O68, and PCR-Based Serogrouping

**DOI:** 10.3390/bios5010051

**Published:** 2015-02-05

**Authors:** Yanhong Liu, Xianghe Yan, Chitrita DebRoy, Pina M. Fratamico, David S. Needleman, Robert W. Li, Wei Wang, Liliana Losada, Lauren Brinkac, Diana Radune, Magaly Toro, Narasimha Hegde, Jianghong Meng

**Affiliations:** 1 Molecular Characterization of Foodborne Pathogens Research Unit, Eastern Regional Research Center, Agricultural Research Service, U.S. Department of Agriculture, Wyndmoor, PA 19038, USA; E-Mails: xianghe.yan@ars.usda.gov (X.Y.); pina.fratamico@ars.usda.gov (P.M.F.); david.needleman@ars.usda.gov (D.S.N.); 2 *E. coli* Reference Center, Department of Veterinary and Biomedical Sciences, The Pennsylvania State University, University Park, PA 16802, USA; E-Mails: rcd3@psu.edu (C.D.); nvh1@psu.edu (N.H.); 3 Animal Genomics and Improvement Laboratory, Agricultural Research Service, U.S. Department of Agriculture, Beltsville, MD 20705, USA; E-Mail: robert.li@ars.usda.gov; 4 J. Craig Venter Institute, Rockville, MD 20850, USA; E-Mails: wwang@jcvi.org (W.W.); llosada@jcvi.org (L.L.); lbrinkac@jcvi.org (L.B.); diana.radune@gmail.com (D.R.); 5 Department of Nutrition & Food Science, University of Maryland, College Park, MD 20742, USA; E-Mails: magaly.toro.i@gmail.com (M.T.); Jmeng@umd.edu (J.M.)

**Keywords:** PCR, *Escherichia coli*, serogroups, DNA sequence, O-antigen gene cluster, detection, identification

## Abstract

The DNA sequence of the O-antigen gene clusters of *Escherichia coli* serogroups O62, O68, O131, O140, O142, and O163 was determined, and primers based on the *wzx* (O-antigen flippase) and/or *wzy* (O-antigen polymerase) genes within the O-antigen gene clusters were designed and used in PCR assays to identify each serogroup. Specificity was tested with *E. coli* reference strains, field isolates belonging to the target serogroups, and non-*E. coli* bacteria. The PCR assays were highly specific for the respective serogroups; however, the PCR assay targeting the O62 *wzx* gene reacted positively with strains belonging to *E. coli* O68, which was determined by serotyping. Analysis of the O-antigen gene cluster sequences of serogroups O62 and O68 reference strains showed that they were 94% identical at the nucleotide level, although O62 contained an insertion sequence (IS) element located between the *rmlA* and *rmlC* genes within the O-antigen gene cluster. A PCR assay targeting the *rmlA* and *rmlC* genes flanking the IS element was used to differentiate O62 and O68 serogroups. The PCR assays developed in this study can be used for the detection and identification of *E. coli* O62/O68, O131, O140, O142, and O163 strains isolated from different sources.

## 1. Introduction

In *Escherichia coli* and other Gram-negative bacteria*,* the major component of the outer membrane is lipopolysaccharide, which consists of three components: lipid A embedded in the membrane, an oligosaccharide core, and the lateral polysaccharide O-antigen. The O-antigen confers antigenic variability to the bacteria due to differences in the sugar components, the linkages, and the structure of the repeat O-units. Traditional serotyping of *E. coli* is based on agglutination reactions of the bacteria with antisera raised in rabbits immunized with different O-group reference strains. The test is performed in tubes, 96-well plates, or on slides [1,2]. The *E. coli* O-antigen is released by heating the bacteria for 2 h at 100 °C, and agglutination or clumping occurs when the O-antigen reacts with its specific antiserum. However, if the *E. coli* is capsulated or rough (does not carry O-antigen), agglutination does not occur. Furthermore, cross-reactions may occur with other *E. coli* O-groups, resulting in equivocal results, and serotyping is generally only performed in a few reference laboratories that have antisera against all of the *E. coli* O-groups. There are currently over 184 different *E. coli* O-groups classified as O1–O187 except for six (O31, O47, O67, O72, O94, O122) that have not been designated. The O-group defines the serogroup, and the combination of the O-antigen and the H-flagellar antigen defines the *E. coli* serotype.

Genes required for synthesis of the *E. coli* O-antigen are located on the chromosomal O-antigen gene cluster, which is located between a conserved 39-bp JUMPstart sequence (upstream) and downstream by the *gnd* gene that encodes for 6-phosphogluconate dehydrogenase [3,4]. Due to the differences in the composition of the O-antigens, the genes that encode for enzymes required for O-antigen synthesis vary among the different *E. coli* serogroups. Many *E. coli* O-antigen gene clusters have been sequenced, and the information has been deposited in GenBank. The sequence information can be used to identify unique regions that can be targeted, for example by PCR assays or other DNA-based methods, to identify the *E. coli* O-group. Furthermore, the sequence information can be used to study the evolution of *E. coli* O-antigens that can occur through gene deletion, acquisition, or inactivation [5].

Genes found in the O-antigen gene clusters that show genetic variability among the different serogroups include the *wzx* (O antigen flippase) and *wzy* (O antigen polymerase) genes, and PCR assays targeting these genes have been developed to identify different *E. coli* serogroups [4,6,7,8,9]. A DNA array approach was developed to identify *E. coli* O-groups using either representative oligonucleotides or PCR products to spot the array and labeled long PCR products for hybridization [10]. Lin *et al.* [11] performed PCR assays targeting the *wzx* and *wzy* genes of ten Shiga toxin-producing *E. coli* (STEC) serogroups, and then used the Luminex system to identify the ten serogroups through binding of the PCR products to fluorescent microspheres conjugated to specific DNA probes for each of the ten serogroups. Furthermore, multiplex assays can be designed to detect specific pathogenic *E. coli* serogroups targeting O-antigen gene cluster sequences and virulence genes [7,12]. Use of the Luminex system (Luminex, Austin, TX, USA) employing monoclonal antibodies coated to carboxylated magnetic microbeads to simultaneously detect Shiga toxin serogroup O157, as well as Shiga toxin 1 and Shiga toxin 2 has also been reported [13]. A review by DebRoy *et al.* [7] provides information on *E. coli* O-antigen gene clusters and methods used for O-group determination.

There are a number of *E. coli* pathotypes, consisting of various *E. coli* O-groups, that have been isolated from animals and that can cause illness in humans and animals. Enteropathogenic *E. coli* (EPEC) O142 has been isolated from infant stools, patients with diarrhea, and piglets [14,15,16]. Shiga toxin-producing *E. coli* (STEC) O62 was isolated from pork, and this serogroup has also been described as an enteroaggregative *E. coli* [17,18]. Verocytotoxin producing *E. coli* (VTEC, also known as STEC) O163 has been associated with cases of hemolytic uremic syndrome [19,20]. In addition, *E. coli* O163 was isolated from animals, including cows [21], lamb [22], goats, sheep [23], and pigs [24]. *E. coli* O131 was associated with pigs with post-weaning diarrhea in China [25], and *E. coli* O140 was associated with broiler chickens with dermatitis [26] and piglets with diarrhea [27].

Various molecular serotyping approaches could be used to identify *E. coli* O-groups, including the use of the Luminex^®^ system, DNA microarrays, or the BioMark^TM^ real-time PCR array system (Fluidigm Corporation, South San Francisco, CA, USA), and others. Using some of these approaches, O-group determination could be coupled with simultaneous identification of virulence genes specific for certain *E. coli* pathotypes. However, to accomplish this, definitive determination of the O-antigen gene cluster sequences of all of the *E. coli* O-groups and of strains identified as untypeable by serotyping is needed. The objectives of this study were to determine the DNA sequence of the O-antigen gene clusters of *E. coli* serogroups O62, O68, O131, O140, O142, and O163, analyze the sequence data, and identify unique regions that are suitable targets for PCR assays to identify these serogroups. This work provides essential information for the application of molecular methods to differentiate *E. coli* serogroups, which is critically needed for accurate identification of *E. coli* and for epidemiological investigations of disease outbreaks.

## 2. Experimental Section

### 2.1. Bacterial Strains and Culture Conditions

*E. coli* O62 (F 10524-41, K-:H30), O68 (P 7d, K-:H4), O131 (S 239, K-:H26), O140 (CDC 149-51, K-:H43), O142 (C 771, H6) and O163 (SN3B/1, K-:H19) reference standard strains were obtained from the World Health Organization [1]. These strains were used for DNA sequencing of the O-antigen gene clusters. Bacterial strains used to validate the specificity of the PCR assays were from the culture collection of the *E. coli* Reference Center at the Pennsylvania State University. The following strains were included in the PCR assays: 148 field strains from *E. coli* serogroups O62, O68, O131, O140, O142, and O163 isolated from humans, animals, food, and water, and 174 *E. coli* standard reference strains belonging to serogroups O1-O187, but excluding O31, O47, O67, O72, O94, and O122, since these serogroups have not been designated [1]. In addition, 16 strains representative of other bacterial genera used to test the specificity of the PCR assays included *Staphylococcus aureus* ATCC13709, *Staphylococcus aureus* ATCC 29213, *Klebsiella pneumoniae* ATCC 27736, *Serratia marcescens* ATCC 13880, *Shigella boydii* ECRC 15.0055, *Salmonella enterica sv.* Typhi ECRC 15.0056, *Enterobacter cloacae* ECRC 15.0057, *Salmonella enterica sv.* Arizonae ECRC15.0058, *Salmonella enterica sv.* Choleraesuis ATCC 14028, *Salmonella enterica sv.* Choleraesuis ATCC 51741, *Salmonella enterica sv.* Anatum ATCC 9270, *Citrobacter freundii* ATCC 8090, *Hafnia alvei* ATCC 29926, *Shigella flexneri* ECRC 15.0059, *Yersinia enterocolitica* ECRC 15.0060, *and Listeria innocua* ATCC 51742*.* All of the bacteria were grown in Luria Bertani (LB) broth or on LB agar plates at 37 °C.

### 2.2. DNA Sequencing and Gene Annotation

Genomic DNA was isolated using the DNeasy Tissue Kit (Qiagen Inc., Valencia, CA, USA) according to the manufacturer’s instructions. Long PCR assays were performed to amplify the O-antigen gene clusters using the Expand Long Template PCR system (Roche Applied Science, Mannheim, Germany) and the JUMPSTART (named for Just Upstream of Many Polysaccharide-associated gene STARTs) and GND (6-phosphogluconate dehydrogenase gene) primer set targeting sequences that flank the *E. coli* O-antigen gene clusters as described previously [12]. However, some modifications were made to the JUMPSTART and GND primer sequences, and they are the following: JUMPSTART primer 5*'*-CATGGTAGCTGTAAAGCCAGGGGCGGTAGCGTG-3*'*; GND primer 5*'*-CATGCTGCCATACCGACGACGCCGATCTGTTGCTTKGACA-3*'* (Integrated DNA Technologies, Coralville, IA, USA). The long PCR conditions were as described previously [9]. The long PCR products were verified on 0.8% agarose gels and purified according to instructions in the QIAquick PCR Purification Kit (Qiagen Inc., Valencia, CA, USA). The long PCR products were sequenced by the methods described below.

DNA integrity was verified using a Bioanalyzer 2100 (Agilent, Palo Alto, CA, USA), and DNA concentration was quantified using a QuantiFluor fluorometer (Promega, Madison, WI, USA). For sequencing with the Roche/454 GS FLX instrument (Roche, 454 Life Sciences, Branford, CT, USA), the O-antigens were amplified from 40 ng of genomic DNA isolated as described above, with eight-bp sample-specific bar coded primers using 2.5 units of AccuPrime Taq DNA Polymerase High Fidelity (Invitrogen, Carlsbad, CA, USA) in a 50-μL reaction buffer containing 200 nM primers, 200 nM dNTP, 60 mM Tris-SO_4_, 18 mM (NH_4_)_2_SO_4_, 2.0 mM MgSO_4_, 1% glycerol, and 100 ng/µL bovine serum albumin (New England BioLabs, Ipswich, MA, USA). PCR was performed using the following cycling profile: initial denaturing at 95 °C for two min followed by 25 cycles of 95 °C 30 s, 50 °C 30 s, and 72 °C 120 s. Bar-coded amplicons were generated from each sample separately, purified using an Agencourt AMPure XP kit (Beckman Coulter Genomics, Danvers, MA, USA), and quantified using a QuantiFluor fluorometer. Bar-coded amplicons from individual samples were pooled in equal mass (molar) ratios. The purified bar-coded amplicon library was further verified and quantified using a BioAnalyzer 2100 (Agilent) and subjected to genome sequencing using the Roche/454 GS FLX. Illumina HiSeq 2000 (San Diego, CA, USA) sequencing was performed as described by Djikeng *et al.* [28] using long PCR products. The sequence reads generated from the Illumina, Roche/454 GS FLX, and the Sanger sequencing method using the 3730 DNA Analyzer (Applied Biosystems, Foster City, CA, USA) (see below) were each first assembled separately. The sequence data and the generated contigs were then combined and assembled into the final O-antigen clusters using CLC Genomics Workbench 4.6.1 (CLC bio, Aarhus, Denmark) and Sequencher version 5.1 software (Gene Codes Corporation, Ann Arbor, MI, USA). Some additional details on the sequencing strategy and contig assembly were as described by Djikeng *et al.* [28]. To confirm the sequences of each of the O-antigen gene clusters, the long PCR products were resequenced using a 3730 DNA Analyzer (Applied Biosystems, Foster City, CA, USA) using primers designed from different regions along the gene clusters, and gene annotation was performed as described previously [9]. The HMMTOP program [29] was used to identify potential transmembrane helices from the amino acid sequences.

### 2.3. PCR Specificity Testing

*E. coli* reference strains [1] and field strains belonging to serogroups O62, O68, O131, O140, O142 and O163, and non-*E. coli* bacteria were grown overnight on tryptic soy agar (TSA) plates at 37 °C. Single colonies were picked and resuspended in 100 µL of Tris-EDTA buffer (pH 8.0) and heated at 100 °C for 10 min. The suspension was centrifuged at 10,000× *g*, and the supernatant containing genomic DNA was used for the PCR reactions.

The PCR primers (Table 1) were designed from the *wzx*, *wzy*, and *rmlA/rmlC* region of the targeted O-serogroups. The PCR reaction mix (20 µL total volume) was comprised of template DNA (1 µL), 300 nM of each primer, and 10 µL of the Power SYBR^®^ Green PCR master mix containing Taq Polymerase (Life Technologies, Carlsbad, CA, USA). RT-PCR reactions were conducted using an AB 7300 Real-Time PCR system (Applied Biosystems). The PCR cycling conditions consisted of an initial denaturation for 10 min at 95 °C followed by 40 cycles at 95 °C for 15 s and 60 °C for 1 min. Reaction mixtures without template DNA and without primers served as negative controls. Data were analyzed using 7300 system SDS software (Applied Biosystems, Foster City, CA, USA).

**Table 1:** PCR primers targeting the *wzx*, *wzy* and *rmlA/C* genes of *E. coli* O62, O68, O131, O140, O142 and O163.

Target Gene	Sequence	Amplicon Size (bp)
O62/O68 *wzx*	F: 5*'* ATGCTGCATTAGCGTTAGCA 3*'*	288
	R: 5*'* CCTGTTGAATTGGCACGTAA 3*'*	
O131 *wzx*	F: 5*'* TCGTGAGAAGGCTTTTTGGT 3*'*	290
	R: 5*'* CCCTATCCAATGCGCTTAAA 3*'*	
O140 *wzx*	F: 5*'* TTGGATAGCCGCGTTAATTC 3*'*	294
	R: 5*'* GCCTGAGTTAGCGGATTGAG 3*'*	
O142 *wzx*	F: 5*'* TCTCCATCCCCGTTTATTTG 3*'*	285
	R: 5*'* CCCCAAACATTAGCATTCGT 3*'*	
O163 *wzy*	F: 5*'* GCAATCTTGAAGCCAGAACC 3*'*	262
	R: 5*'* GATAAACCCAGCCACCAAA 3*'*	
O62/O68 *rmlA/C*	F: 5*'* CTACACTGATGTTAGCGGGTATT 3*'*	1969 (for O62)
	R: 5*'* CCGCTTCAAATTCAGGACAATAA 3*'*	1172 (for O68)

### 2.4. Nucleotide Sequence Accession Numbers

DNA sequences of the O-antigen gene clusters of *E. coli* O62, O68, O131, O140, O142 and O163 were deposited into GenBank with the following accession numbers: JX501334, KJ534585, JX501336, JX501338, JX501337, and JX501339, respectively.

## 3. Results and Discussion

**Figure 1:**
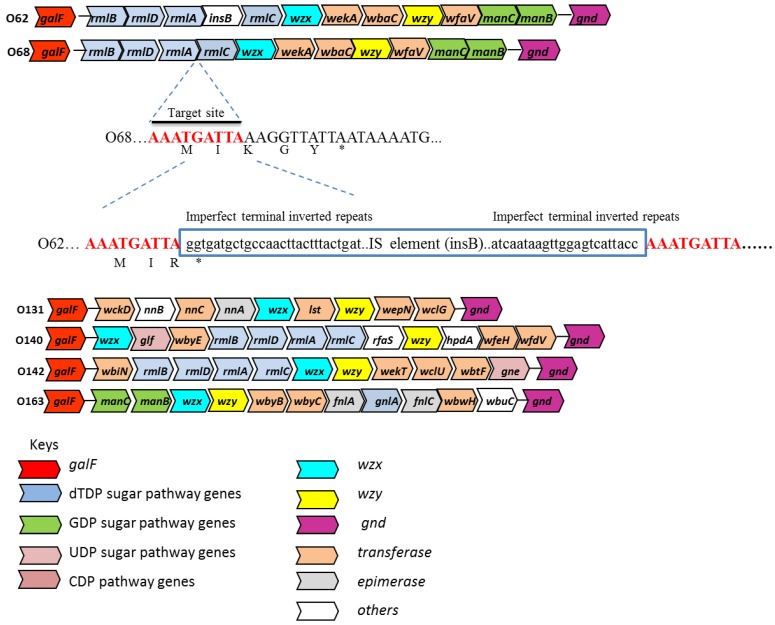
Organization of the O-antigen gene clusters for *E. coli* O62 O68, O131, O140, O142 and O163. The insertion sequence (IS) insertion and its flanking sequences in *E. coli* O62 is shown relative to the O68 O-antigen gene cluster. The arrows represent the location and direction of translation for putative genes in the clusters. The genes are not drawn in scale. The 9-bp target duplications are shown in red. The 23-bp imperfect terminal repeats in the IS element (boxed) are shown in bold. Deduced amino acids sequences (shown in one-letter code) of the IS insertion site are also shown. *galF* is known to be upstream of the O-antigen gene clusters [7].

DNA sequences obtained from the *E. coli* O antigen gene clusters of serogroup O62, O68, O131, O140, O142 and O163 contained 9 to 12 ORFs (Figure 1 and Appendix Table A1, Tables A2, Tables A3, Tables A4, Tables A5 and Tables A6), all in the same transcriptional direction from *galF* to *gnd.* The deduced amino acid sequences from these ORFs were used to search the NCBI database for an indication of their possible functions. Gene names were assigned on the basis of the Bacterial Polysaccharide Gene Nomenclature system (http://sydney.edu.au/science/molecular_bioscience/BPGD/).

### 3.1. Sequence Analysis of the E. coli O-Antigen Gene Clusters of Serogroups O62, O68, O131, O140, O142 and O163

The genes coding for proteins within the *E. coli* O-antigen gene clusters primarily consist of three categories: nucleotide sugar biosynthesis, glycosyl transferase, and O-antigen processing. Nucleotide sugar biosynthesis genes encode for proteins that are involved in the synthesis of the nucleotide sugar precursors of the O-antigen, which occurs in the cytoplasm. Genes coding for glycosyl transferases are responsible for transferring the various precursor sugars to form an oligosaccharide on a carrier lipid, undecaprenyl phosphate (UndP), which is located on the inner membrane facing the cytoplasmic side. The O-antigen processing proteins include a flippase (Wzx) and the O-antigen polymerase (Wzy). These proteins are involved in translocation of the O-units across the membrane and in O-antigen polymerization, respectively. The O-antigen is synthesized by sequential transfer of sugars and other components to the first sugar, which is then translocated and flipped across the membrane by Wzx. They are further polymerized by Wzy. Both Wzx and Wzy are hydrophobic proteins with transmembrane helices that show high variation in sequence among different microorganisms [7].

#### 3.1.1. Sugar Biosynthetic Pathway Genes

The four genes involved in the biosynthesis of dTDP-L-rhamnose [30] are clustered together in O68, O140 and O142 in the gene order of *rmlBDAC* (Appendix Tables A2, Tables A4, Tables A5). The *rmlB* (dTDP-glucose 4,6-dehydratase), *rmlD* (dTDP-4-dehydrorhamnose reductase), *rmlA* (glucose-1-phosphate thymidylyltransferase), *rmlC* (dTDP-4-dehydrorhamnose 3,5-epimerase) genes are also present in O62 with the same gene order except there is a transposase (*insB*) between the *rmlA* and *rmlC* genes (Table 2). The two genes (*fnlA* and *fnlC*) involved in the biosynthesis of UDP-L-FucNAc are present in the O-antigen gene cluster of *E. coli* O163 (Appendix Tables A6). The *fnlA* (UDP-glucose 4-epimerase) and *fnlC* (UDP-N-acetylglucosamine 2-epimerase) genes are present in several other reported gene clusters coding for UDP-L-FucNAc containing structures [30]. The *manB* and *manC* genes present in the O antigen gene clusters of O62, O68 (Appendix Tables A1 and Tables A2), and O163 (Appendix Tables A6) have been identified to be responsible for the biosynthesis of GDP-D-mannose [30]. *manB* and *manC* encode phosphomannomutase and mannose-1-phosphate guanyltransferase, respectively.

The polysaccharide structure of the *E. coli* O142 and O68 O-antigens has been determined [31,32,33]. The proposed function of the genes in the O-antigen gene clusters of *E. coli* O142 and O68 correlates well to the identified O142 and O68 polysaccharide structure [32,33].

#### 3.1.2. Sugar Transferase Genes

Genes encoding for sugar transferases were identified based on their similarity to known sugar transferases. As shown in Figure 1 and Appendix Tables A1, Tables A2, Tables A3, Tables A4, Tables A5 and Tables A6, O62, O68, O140, and O163 each contained three sugar transferases, whereas O131 and O142 contained five and four sugar transferases, respectively. These ORFs have a high degree of sequence variation (30%–60% amino acid similarity), which is consistent with previous studies [30].

#### 3.1.3. O Antigen Processing Genes

All of the six O-antigen gene clusters contained the *wzx* and *wzy* genes located in different regions within the gene clusters (Appendix Tables A1, Tables A2, Tables A3, Tables A4, Tables A5 and Tables A6). Analysis using the HMMTOP program [29] indicated that all six Wzx proteins contained 12 transmembrane helices, whereas the Wzy proteins contained 10 transmembrane helices, with the exception of the Wzy protein from O142 that contained 13 transmembrane helices.

### 3.2. Development of PCR Assays to Identify E. coli O62/O68, O131, O140, O142, and O163 Serogoups

Primers were designed targeting the *wzx* and/or *wzy* genes from the above *E. coli* serogroups (Table 1), and they were used in PCR assays to determine specificity for each serogroup against 174 *E. coli* standard strains, as well as field *E. coli* strains serotyped as O62, O68, O131, O140, O142 and O163 isolated from humans, animals, food, or water. Sixteen non-*E. coli* strains (see Experimental Section for the list of non-*E. coli* strains) were also included as negative controls for specificity testing. PCR assays targeting the *wzx/wzy* genes showed high specificity for each serogroup with no amplification of *wzx/wzy* genes from other *E. coli* serogroups and no amplification of DNA of other bacterial genera. All of the field isolates serogrouped as *E. coli* O131, O140, O142 and O163 were positive by PCR for the corresponding serogroup with 100% accuracy. However, the O62 *wzx* PCR assay also gave a positive result with the O68 reference strain (Table 2). This is not surprising, since our sequencing data alsodemonstrated that the *wzx* sequences of O62 were identical with those of O68 (Appendix Tables A1 and Tables A2). The field strains of *E. coli* O62 (*n* = 2) and O68 (*n* = 6) also exhibited positive PCR results with the *wzx* primers of O62.

**Table 2:** Specificity of the PCR assays for O groups tested.

O Group Tested	Strains Tested	Specificity
O62/O68 (*wzx*) PCR	Reference strains (O1–O181)	All negative except O62 and O68 positive control strains
	O62 field isolates (*n* = 2)	2/2 positive (100%) ^a^
	O68 field isolates (*n* = 6)	6/6 positive (100%) ^a^
	non-*E. coli* (*n* = 16)	100% negative
O131 (*wzx*) PCR	Reference strains (O1–O181)	All negative except O131 positive control strain
	O131 field isolates (*n* = 15)	15 positive (100%)
	non-*E. coli* (*n* = 16)	100% negative
O140 (*wzx*) PCR	Reference strains (O1–O181)	All negative except O140 positive control strain
	O140 field isolates (*n* = 28)	28 positive (100%)
	non-*E. coli* (*n* = 16)	100% negative
O142 (*wzx*) PCR	Reference strains (O1–O181)	All negative except O142 positive control strain
	O142 field isolates (n = 50)	50 positive (100%)
	Non-*E. coli* (n = 16)	100% negative
O163 (*wzy*) PCR	Reference strains (O1–O181)	All negative except O163 positive control strain
	O163 field isolates (*n* = 47)	47 positive (100%)
	non-*E. coli* (*n* = 16)	100% negative

^a^ Although two strains were positive using both O62 and O68 antisera similar to the O62 reference strain, one strain did not show the presence of the insertion element found in the O62 reference strain by PCR, therefore, one strain could be either O62 or O68.

### 3.3. Acquisition of the IS1 Element in *E. coli* O62 and Evolutionary Implications and Differentiation of Serogroups O62 and O68

Analysis of the O-antigen gene clusters of *E. coli* O62 and O68 showed that they are almost identical, except that *E. coli* O62 contained an IS element insertion (ORF4), 748 bp in size at the end of the *rmlA* gene. ORF4 (*insB*) encodes for a transposase that is identical to IS1 transposition proteins in *Shigella flexneri* 2b (Appendix Table A1). The IS1 element in *E. coli* O62 is inserted within the third codon from the end of the *rmlA* gene, resulting in a truncated protein ending with an R (arginine) in place of K (lysine), and in comparison with *E. coli* O68 the last two amino acids are missing (Figure 1). IS1 is a common mobile genetic element that usually generates a 8 to 9-bp target duplication upon integration [34]. In addition, the IS1 element contains 23-bp imperfect terminal repeats that is a characteristic of an IS element [35,36]. The IS1 element is widely distributed in prokaryotic genomes, is highly mobile, and can be a source of genome rearrangements [37,38]. The IS elements present in *E. coli* O24 seemed to play important roles for the assembly of the O24 O-antigen gene cluster by mediating lateral gene transfer and gene inactivation [5]. The high level of similarity between the O-antigen gene clusters of the *E. coli* O62 and O68 reference strains suggests that the O-antigen gene clusters are very closely related and may be derived from a common ancestor.

To differentiate *E. coli* O62 and O68, primers were designed targeting the *rmlA* and *rmlC* region flanking the IS element from O62 (Table 1). The predicted PCR products for O62 and O68 are 1969 bp and 1172 bp, respectively. These primers were used in PCR assays to differentiate two O62 and six O68 (determined by serotyping) field strains in our strain collection. Of the strains tested, six O68 strains were positive for O68 using the *rmlA/C* PCR (*i.e.*, lacked the IS element), and they were positive only for O68 by serotyping (Table 3). One of the two strains that were originally serotyped as O62 strains was positive for O68 according to the PCR assay targeting *rmlA/C* (*i.e.*, lacked the IS element) (Table 3); however, this strain was also positive for O68 by serotyping, similar to the pattern for O62 strains, which are positive by serotyping for both O62 and O68. Therefore, this strain should either be re-assigned as a variant of O68, or it is possible that it is actually O62, but does not carry the IS element. Because there are so few field strains belonging to serogroup O62 in a collection of approximately 70,000 strains at the *E. coli* Reference Center at the Pennsylvania State University, collected over the last fifty years, this suggests that this O-group is not commonly found in animals, humans, and the environment. Our data show that the O-antigen gene clusters of *E. coli* O62 and O68 are very similar. The high similarities between O62 and O68 likely result in antisera cross reaction, which is an important problem in traditional serotyping. It is puzzling, however, the antiserum prepared against O62 does not cross react with O68, whereas antiserum against O68 cross reacts with O62. To accurately serotype O62/68 strains, it is important to first perform serotyping followed by the PCR assay using the *rmlA/C* primers flanking the IS element for O62 positive (by serotyping) strains.

**Table 3:** Serotyping results and PCR using *rmlA/C* primers for O62/O68 field strains.

O62/68 Field Strain Designation (ECRC#) ^a^	Serotyping Using O62 Antiserum	Serotyping Using O68 Antiserum	Serogroup by PCR Using *rmlA/C* Primers
12.0591	Positive	Positive	O62 (1969 bp)
94.0296	Positive	Positive	O68/O62 (1172 bp) ^b^
1.2557	Negative	Positive	O68
3.1263	Negative	Positive	O68
4.0175	Negative	Positive	O68
4.2378	Negative	Positive	O68
5.1791	Negative	Positive	O68
6.2334	Negative	Positive	O68

^a^ ECRC#—*E. coli* Reference Center strain designation; ^b^ Although this strain was positive using both O62 and O68 antisera similar to O62 strain 12.0591 and the O62 reference strain, it did not show the presence of the insertion element by PCR.

## 4. Conclusions

The O-antigen gene cluster sequences for six *E. coli* serogroups have been determined, and thus PCR primers can be designed for unique regions within the gene cluster sequences to develop genetic-based methods for serotyping, which are more specific than traditional serotyping. The PCR assays designed in the current study could potentially be used for rapid diagnostic screening for the *E. coli* serogroups. Since serotyping results are often ambiguous and sometimes may not be able to distinguish the serogroups, PCR assays in conjunction with serotyping may be able to circumvent these problems and distinguish the serogroups more accurately.

## Appendix

**Table A1:** Open reading frames (ORFs) in the O-antigen gene cluster of *E. coli* serogroup O62.

ORF	Proposed Gene Name	Location	No. of Amino Acids	Putative Function	Most Significant Similarity (Accession No.)	% Amino Acid Identity/% Similarity
1	*rmlB*	106:1191	361	dTDP-glucose 4,6-dehydratase	dTDP-glucose 4,6-dehydratase [*Escherichia coli*] Sequence ID: ref|WP_001723646.1|	99/100
2	*rmlD*	1191:2090	299	glucose-1-phosphate thymidylyltransferase	dTDP-4-dehydrorhamnose reductase [*Escherichia coli*] Sequence ID: ref|WP_001723645.1|	100/100
3	*rmlA*	2148:3020	290	glucose-1-phosphate thymidylyltransferase	glucose-1-phosphate thymidylyltransferase [*Escherichia coli*] Sequence ID: ref|WP_021516244.1|	99/100
4	*insB*	3265:3768	167	Transposases IS1	IS1 transposition protein [*Shigella flexneri* 2b] Sequence ID: ref|NP_052905.1|	100/100
5	*rmlC*	3808:4350	180	dTDP-4-dehydrorhamnose 3,5-epimerase	dTDP-4-dehydrorhamnose 3,5-epimerase [*Escherichia coli*] Sequence ID: ref|WP_001723643.1|	100/100
6	*wzx*	4365:5576	403	O antigen flippase	polysaccharide biosynthesis family protein [*Escherichia coli*] Sequence ID: ref|WP_001723642.1|	100/100
7	*wekA*	5584:6534	316	glycosyl transferase	hypothetical protein [*Escherichia coli*] Sequence ID: ref|WP_001607665.1|	100/100
8	*wbcC*	6515:7603	362	glycosyl transferase	hypothetical protein [*Escherichia coli*] Sequence ID: ref|WP_001607663.1|	99/100
9	*wzy*	7593:8711	372	O antigen polymerase	putative membrane protein [*Escherichia coli*] Sequence ID: ref|WP_001723640.1|	99/100
10	*wfaV*	8713:9894	393	glycosyl transferases group 1 family	glycosyl transferases group 1 family protein [*Escherichia coli*] Sequence ID: ref|WP_001723639.1|	99/99
11	*manC*	9891:11315	474	mannose-1-phosphate guanyltransferase	mannose-1-phosphate guanylyltransferase/mannose-6-phosphate isomerase [*Escherichia coli*] Sequence ID: ref|WP_001723638.1|	99/99
12	*manB*	11405:12775	456	phosphomannomutase	phosphoglucomutase/phosphomannomutase, C-terminal domain protein [*Escherichia coli*] Sequence ID: ref|WP_001723637.1|	99/100

**Table A2:** Open reading frames (ORFs) in the O-antigen gene cluster of *E. coli* serogroup O68.

ORF	Proposed Gene Name	Location	No. of Amino Acids	Putative Function	Most Significant Similarity (Accession No.)	% Amino Acid Identity/% Similarity
1	*rmlB*	106:1191	361	dTDP-glucose 4,6-dehydratase	dTDP-glucose 4,6-dehydratase [*Escherichia coli*] Sequence ID: ref|WP_001723646.1|	99/100
2	*rmlD*	1191:2090	299	glucose-1-phosphate thymidylyltransferase	dTDP-4-dehydrorhamnose reductase [*Escherichia coli*] Sequence ID: ref|WP_001723645.1|	100/100
3	*rmlA*	2148:3026	292	glucose-1-phosphate thymidylyltransferase	glucose-1-phosphate thymidylyltransferase [*Escherichia coli*] Sequence ID: ref|WP_021516244.1|	99/100
4	*rmlC*	3031:3573	180	dTDP-4-dehydrorhamnose 3,5-epimerase	dTDP-4-dehydrorhamnose 3,5-epimerase [*Escherichia coli*] Sequence ID: ref|WP_001723643.1|	100/100
5	*wzx*	3588:4799	403	O antigen flippase	polysaccharide biosynthesis family protein [*Escherichia coli*] Sequence ID: ref|WP_001723642.1|	100/100
6	*wekA*	4807:5757	316	glycosyl transferase	hypothetical protein [*Escherichia coli*] Sequence ID: ref|WP_001607665.1|	100/100
7	*wbcC*	5738:6826	362	glycosyl transferase	hypothetical protein [*Escherichia coli*] ref|WP_001607663.1|	99/100
8	*wzy*	6816:7934	372	O antigen polymerase	putative membrane protein [*Escherichia coli*] Sequence ID: ref|WP_001723640.1|	99/100
9	*wfaV*	7936:9117	393	glycosyl transferases group 1 family	glycosyl transferases group 1 family protein [*Escherichia coli*] Sequence ID: ref|WP_001723639.1|	100/100
10	*manC*	9114:10538	474	mannose-1-phosphate guanyltransferase	mannose-1-phosphate guanylyltransferase/mannose-6-phosphate isomerase [*Escherichia coli*] Sequence ID: ref|WP_001723638.1|	100/100
11	*manB*	10628:11998	456	phosphomannomutase	phosphoglucomutase/phosphomannomutase, C-terminal domain protein [*Escherichia coli*] Sequence ID: ref|WP_001723637.1|	99/100

**Table A3:** Open reading frames (ORFs) in the O-antigen gene cluster of *E. coli* serogroup O131.

ORF	Proposed Gene Name	Location	No. of Amino Acids	Putative Function	Most Significant Similarity (Accession No.)	% Amino Acid Identity/% Similarity
1	*wckD*	589–1209	206	sialic acid O-acetyltransferase NeuD family sugar O-acyltransferase	ref|YP_002403330.1| WckD [*Escherichia coli* 55989]	91/98
2	*nnaB*	1211–2251	346	N-acetylneuraminic acid synthetase	ref|ZP_02904231.1| NnaB [*Escherichia albertii* TW07627]	94/97
3	*nnaC*	2254–3516	420	N-acylneuraminate cytidylyltransferase	ref|ZP_02904216.1| N-acylneuraminate cytidylyltransferase [*Escherichia albertii* TW07627]	90/96
4	*nnaA*	3513–4694	393	UDP-N-acetylglucosamine 2-epimerase	ref|ZP_02904222.1| UDP-N-acetylglucosamine 2-epimerase [*Escherichia albertii* TW07627]	91/95
5	*wzx*	4691–5959	422	O antigen flippase	ref|ZP_02904256.1| Lsg [*Escherichia albertii* TW07627]	87/93
6	*lst*	5966–6940	324	UDP-glucose:glucosyl LPS a1,2-glucosyltransferase	ref|ZP_02904182.1| putative Lst [*Escherichia albertii* TW07627]	79/88
7	*wzy*	7024–8265	413	O antigen polymerase	ref|YP_002310938.1| unnamed protein product [*Shewanella piezotolerans* WP3]	31/52
8	*wepN*	8262–9122	286	glycotransferase	ref|ZP_03611741.1| hypothetical protein AM202_0156 [*Actinobacillus minor* 202]	29/52
9	*wclG*	9587–10057	156	glycosyl transferase	ref|ZP_07136721.1| glycosyltransferase, group 2 family protein [*Escherichia coli* MS 115-1]	64/80

**Table A4:** Open reading frames (ORFs) in the O-antigen gene cluster of *E. coli* serogroup O140.

ORF	Proposed Gene Name	Location	No. of Amino Acids	Putative Function	Most Significant Similarity (Accession No.)	% Amino Acid Identity/% Similarity
1	*wzx*	26–1273	415	O antigen flippase	gb|ACA24898.1| Wzx [*Escherichia coli*]	62/82
2	*glf*	1270–2379	369	UDP-galactopyranose mutase	gb|EFZ69214.1| UDP-galactopyranose mutase [*Escherichia coli* OK1357]	73/88
3	*wbyE*	2383–3375	330	group 1 glycosyl transferase	ref|YP_002987178.1| hypothetical protein Dd703_1557 [*Dickeya dadantii* Ech703]	45/63
4	*rmlB*	3420–4505	361	dTDP-glucose 4,6-dehydratase	gb|AAZ85703.1| dTDP-glucose 4,6-dehydratase [*Escherichia coli*]	98/96
5	*rmlD*	4505–5404	299	dTDP-4-dehydrorhamnose reductase	gb|EGB44384.1| RmlD substrate binding domain-containing protein [*Escherichia coli* H120]	99/98
6	*rmlA*	5462–6337	291	glucose-1-phosphate thymidylyltransferase	gb|ABE98410.1| glucose-1-phosphate thymidylyltransferase [*Escherichia coli*]	99/99
7	*rmlC*	6345–6878	177	dTDP-4-dehydrorhamnose 3,5-epimerase	ref|YP_853147.1| rmlC gene product [*Escherichia coli* APEC O1]	85/90
8	*rfaS*	6945–7913	322	lipopolysaccharide core biosynthesis protein	gb|EHN67535.1| lipopolysaccharide core biosynthesis protein [*Comamonas testosteroni* ATCC 11996]	35/57
9	*wzy*	7936–9126	396	O antigen polymerase	gb|ACH97152.1| Wzy [*Escherichia coli*]	26/49
10	*hpdA*	9137–9913	258	unknown	ref|YP_001534197.1| hypothetical protein Dshi_2863 [*Dinoroseobacter shibae* DFL 12]	40/56
11	*wfeH*	10117–10761	214	glycosyl transferase	ref|ZP_06693546.1| predicted protein [*Acinetobacter* sp. SH024]	39/56
12	*wfdV*	10758–11516	252	glycosyl transferase	gb|AEH27518.1| WehL [*Cronobacter muytjensii*]	63/77

**Table A5:** Open reading frames (ORFs) in the O-antigen gene cluster of *E. coli* serogroup O142.

ORF	Proposed Gene Name	Location	No. of Amino Acids	Putative Function	Most Significant Similarity (Accession No.)	% Amino Acid Identity/% Similarity
1	*wbiN*	78–1094	338	glycosyl transferase	ref|YP_002329693.1| *wbiN* gene product [*Escherichia coli* O127:H6 str. E2348/69]	58/71
					ref|ZP_07222371.1| glycosyltransferase, group 1 family [*Escherichia coli* MS 78-1]	
					ref|ZP_10058779.1| hypothetical protein ESBG_00585 [*Escherichia* sp. 4_1_40B]	
2	*rmlB*	1114–2199	361	dTDP-glucose-4,6-dehydratase	ref|YP_002391833.1| dTDP-glucose-4,6-dehydratase [*Escherichia coli* S88]	85/92
3	*rmlD*	2109–3098	329	dTDP-6-deoxy-L-mannose-dehydrogenase	gb|AAZ85704.1| dTDP-6-deoxy-L-mannose-dehydrogenase [*Escherichia coli*]	82/91
4	*rmlA*	3129–4028	299	glucose-1-phosphate thymidylyltransferase	gb|ABE98410.1| glucose-1-phosphate thymidylyltransferase [*Escherichia coli*]	93/96
5	*rmlC*	4018–4590	190	dTDP-4-dehydrorhamnose 3,5-epimerase	gb|ACA24817.1| RmlC [*Escherichia coli*]	75/85
6	*wzx*	4587–5831	414	O antigen flippase	ref|YP_541307.1| O-antigen transporter [*Escherichia coli* UTI89]	58/79
7	*wzy*	5883–7061	392	O antigen polymerase	gb|ADC54950.1| Wzy [*Escherichia coli*]	49/68
8	*wekT*	7010–7978	322	rhamnosyltransferase	ref|YP_541305.1| rhamnosyltransferase [*Escherichia coli* UTI89]	59/74
9	*wclU*	7975–8763	262	glycosyltransferase	ref|YP_541304.1| glycosyltransferase [*Escherichia coli* UTI89]	56/70
10	*wbtF*	8760–9854	364	glycosyltransferase	gb|AFI60269.1| WepF [*Cronobacter sakazakii*]	53/73
11	*gne*	10098–11117	339	UDP-glucose 4-epimerase	ref|ZP_07142276.1| UDP-glucose 4-epimerase [*Escherichia coli* MS 182–1]	74/86

**Table A6:** Open reading frames (ORFs) in the O-antigen gene cluster of *E. coli* serogroup O163.

ORF	Proposed Gene Name	Location	No. of Amino Acids	Putative Function	Most Significant Similarity (Accession No.)	% Amino Acid Identity/% similarity
1	*manC*	379:1818	479	mannose-1-phosphate guanylyltransferase *manC*	mannose-1-phosphate guanylyltransferase manC [*Enterobacterc loacae*] ref|WP_023300545.1|	82/89
2	*manB*	1925:3325	466	Phosphomanno-mutase	phosphomannomutase [*Enterobacter cloacae subsp. cloacae* ENHKU01] ref|YP_006579401.1|	90/96
3	*wzx*	3318:4571	417	O-antigen repeat unit transporter	hypothetical protein SARI_00795 [*Salmonella enterica* subsp. Arizonaeserovar 62:z4,z23:- str. RSK2980] Sequence ID: ref|YP_001569857.1|	49/55
4	*wzy*	4568:5803	411	O antigen polymerase	O-unit polymerase [*Salmonella enterica* subsp. Arizonae] Sequence ID: gb|ACJ26814.1|	57/66
5	*wbyB*	5815:6828	337	glycosyl transferase, group 1	glycosyl transferase, group 1 [*Rhodopirellula baltica*] Sequence ID: ref|WP_007337184.1|	42/44
6	*wbyC*	6848:7951	367	glycosyl transferase, group 1	hypothetical protein [*Bacillus cereus*] Sequence ID: ref|WP_000651756.1|	40/46
7	*fnlA*	8076:9167	363	UDP-glucose 4-epimerase	hypothetical protein SARI_00798 [*Salmonella enterica* subsp. Arizonae serovar 62:z4,z23:- str. RSK2980] Sequence ID: ref|YP_001569860.1|	87/91
8	*qnlA*	9133:10035	300	dTDP-4-dehydrorhamnose reductase	hypothetical protein SARI_00799 [*Salmonella enterica* subsp. Arizonae serovar 62:z4,z23:- str. RSK2980] Sequence ID: ref|YP_001569861.1|	67/74
9	*fnlC*	10007:11164	385	UDP-N-acetylglucosamine 2-epimerase	UDP-N-acetylglucosamine 2-epimerase [*Salmonella enterica* subsp. Arizonae] Sequence ID: gb|ACJ26818.1|	68/84
10	*wbwH*	11149:12357	402	glycosyl transferase	hypothetical protein SARI_00801 [*Salmonella enterica* subsp. Arizonae serovar 62:z4,z23:- str. RSK2980] Sequence ID: ref|YP_001569863.1|	67/81
11	*wbuC*	12380:12877	165	unknown	conserved LPS biosynthetic protein [*Salmonella enterica* subsp. Arizonae] Sequence ID: gb|ACJ26820.1|	53/75
